# Ethyl 5-amino-1-[(4-methyl­phen­yl)sulfon­yl]-1*H*-pyrazole-4-carboxyl­ate

**DOI:** 10.1107/S1600536813019326

**Published:** 2013-08-03

**Authors:** Abdel-Sattar S. Hamad Elgazwy, Ibrahim F. Nassar, Peter G. Jones

**Affiliations:** a Chemistry Department, Faculty of Science, Ain Shams University, Abbassia 11566, Cairo, Egypt; b Faculty of Education, Ain Shams University, Abbassia, Cairo, Egypt; c Institut für Anorganische und Analytische Chemie, Technische Universität Braunschweig, Postfach 3329, 38023 Braunschweig, Germany

## Abstract

In the title mol­ecule, C_13_H_15_N_3_O_4_S, the benzene and pyrazole rings are inclined to each other at 77.48 (3)°. Two amino H atoms are involved in bifurcated hydrogen bonds, *viz.* intra­molecular N—H⋯O and inter­molecular N—H⋯O(N). The inter­molecular hydrogen bonds link the mol­ecules related by translation in [100] into chains. A short distance of 3.680 (3) Å between the centroids of benzene and pyrazole rings from neighbouring mol­ecules shows the presence of π–π inter­actions, which link the hydrogen-bonded chains into layers parallel to the *ab* plane.

## Related literature

For background details and information on the synthesis, see: Elgazwy, Ismail *et al.* (2012 ▶); Elgazwy, Soliman *et al.* (2012 ▶).
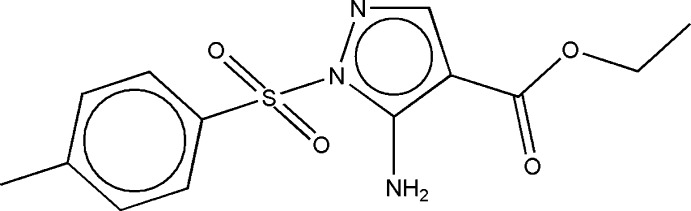


## Experimental

### 

#### Crystal data

C_13_H_15_N_3_O_4_S
*M* *_r_* = 309.34
Monoclinic, 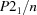

*a* = 6.27869 (7) Å
*b* = 15.43607 (12) Å
*c* = 15.27141 (13) Å
β = 96.2633 (9)°
*V* = 1471.24 (2) Å^3^
*Z* = 4
Cu *K*α radiation
μ = 2.14 mm^−1^
*T* = 100 K
0.25 × 0.12 × 0.10 mm

#### Data collection

Oxford Diffraction Xcalibur (Atlas, Nova) diffractometer
Absorption correction: multi-scan (*CrysAlis PRO*; Agilent, 2012 ▶) *T*_min_ = 0.800, *T*_max_ = 1.000
51079 measured reflections
3043 independent reflections
3035 reflections with *I* > 2σ(*I*)
*R*_int_ = 0.024

#### Refinement

*R*[*F* ^2^ > 2σ(*F* ^2^)] = 0.027
*wR*(*F* ^2^) = 0.074
*S* = 1.05
3043 reflections
201 parameters
H atoms treated by a mixture of independent and constrained refinement
Δρ_max_ = 0.34 e Å^−3^
Δρ_min_ = −0.39 e Å^−3^

### 

Data collection: *CrysAlis PRO* (Agilent, 2012 ▶); cell refinement: *CrysAlis PRO*; data reduction: *CrysAlis PRO*; program(s) used to solve structure: *SHELXS97* (Sheldrick, 2008 ▶); program(s) used to refine structure: *SHELXL97* (Sheldrick, 2008 ▶); molecular graphics: *XP* (Siemens, 1994 ▶); software used to prepare material for publication: *SHELXL97*.

## Supplementary Material

Crystal structure: contains datablock(s) I, global. DOI: 10.1107/S1600536813019326/cv5421sup1.cif

Structure factors: contains datablock(s) I. DOI: 10.1107/S1600536813019326/cv5421Isup2.hkl

Supplementary material file. DOI: 10.1107/S1600536813019326/cv5421Isup3.ps

Supplementary material file. DOI: 10.1107/S1600536813019326/cv5421Isup5.cml

Additional supplementary materials:  crystallographic information; 3D view; checkCIF report

## Figures and Tables

**Table 1:** Hydrogen-bond geometry (Å, °)

*D*—H⋯*A*	*D*—H	H⋯*A*	*D*⋯*A*	*D*—H⋯*A*
N3—H01⋯O1^i^	0.852 (18)	2.554 (17)	3.0631 (13)	119.3 (13)
N3—H01⋯O2	0.852 (18)	2.237 (17)	2.8624 (13)	130.3 (14)
N3—H02⋯O3	0.865 (18)	2.456 (17)	2.9775 (13)	119.4 (13)
N3—H02⋯N2^i^	0.865 (18)	2.206 (18)	3.0216 (14)	157.1 (15)

Symmetry code: (i) 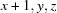.

**Fig. 1.**
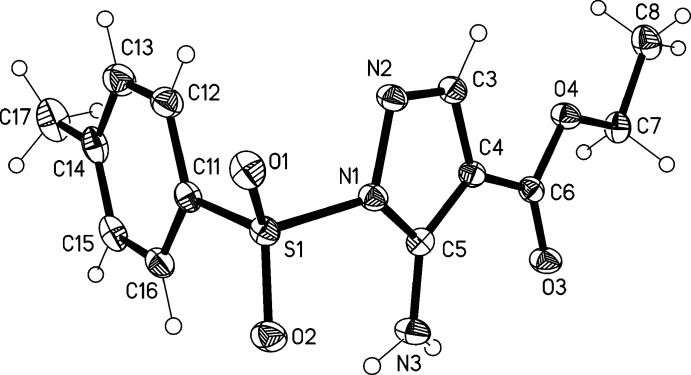
The molecular structure of (I) showing the atomic numbering and 50% probabilty displacement ellipsoids.

## supplementary crystallographic information

### 1. Comment

During the course of our studies directed toward exploring the synthetic potential of 2,3-dihydroprazoles and thiazoles for synthesizing of novel antibacterial agents (Elgazwy, Ismail *et al.*, 2012), we have recently reported various successful approaches for synthesis of 3,5-diaryl-4,5-dihydropyrazole analogues by the reaction of chalcones with thiosemicarbazide or with hydrazine hydrate in the presence of acetic acid (Elgazwy, Soliman *et al.*, 2012). In conjunction with this work, we report here the title compound (I).

In (I) (Fig. 1), the benzene and pyrazole rings form a dihedral angle of 77.48 (3)°. Each amino hydrogen is involved in bifurcated hydrogen bond (one intra- and one intermolecular, Table 1), as was implied by the broad and downfield-shifted peak of the ethyoxy protons in the ^1^H NMR. The intermolecular interactions, for which the acceptors are a tosyl oxygen and a pyrazole nitrogen, link the molecules related by translation parallel to the *a* axis. Short distance of 3.680 (3) Å between the centroids of benzene and pyrazole rings from the neighboring molecules shows a presence of π–π interactions, which link further hydrogen-bonded chains into layers parallel to *ab* plane.

### 2. Experimental

The reaction of unsaturated ketones of (*E*)-ethyl 2-cyano-3-ethoxyacrylate with 4-methylbenzenesulfonohydrazide was conducted in the presence of ethanol at reflux for 16 h. The 4,5-dihydro-1*H*-pyrazole analogues were obtained regiospecifically with satisfactory yields (60–96%). In most cases, N-1 was substituted by a strongly electron-withdrawing group that hindered the elimination of water and a subsequent aromatization of the pyrazole ring. For the title compound, we further employed an ethyl carboxylate at C-4 and an amino group at C-5. Pyrazoles showed sets of ^1^H and ^13^C NMR data that corresponded to the proposed structures. Compound (3) showed ^1^H NMR chemical shifts as a characteristic AB system. The ^13^C NMR spectra showed typical chemical shifts of 4,5-dihydro-1*H*-pyrazole rings on average at δ 157.0 (C-3), 46.4 (C-4), 88.4 (C-5). It is noteworthy that C-5 showed similar chemical shifts for series (3–18), emphasizing the similarity of the inductive effect of amino group.

To a solution of 4-methylbenzenesulfonohydrazide (1.86 g m, 0.01 mol) in absolute ethanol (20 ml) was added (*E*)-ethyl 2-cyano-3-ethoxyacrylate (1.69 g m, 0.01 mol). The reaction mixture was heated at reflux temperature for 16 h. The reaction mixtures were cooled at room temperature and the solvents were evaporated under reduced pressure, the resulting solids were crystallized from ethanol to afford solid (I). Yield 201 mg, 65%; m.p 135–137 °C dec. Diffraction-quality crystals were grown by slow diffusion of ethanol solution. IR (cm^-1^): (NH) 3480, (C═O),1693, (C═N) 1615. ^1^H NMR (300 MHz, DMSO-d6): δ 1.29 (t, 3H, ^1–3^*J*_H,H_ = 7.2, O—CH_2_—C*H*_3_), 2.34 (s, 3H, CH_3_), 4.18 (q, 2H, ^1–3^*J*_H,H_ = 2.7, O—C*H*_2_-CH_3_), 4.22 (s, 2H, –NH_2_), 7.46 (d, 2H, *J* = 8.1 Hz, Ph—H(*m*)), 7.84 (d, 2H, *J* = 8.1 Hz, Ph—H(O)), 7.90 (s, 1H, pyrazole-H) p.p.m.. GC/MS: m/*z* (%) 309 (8.37), 245 (6.07), 199 (5.90), 155 (12.81), 91 (33.88), 63 (91.51),44 (100). Analysis: Calcd. for C_13_H_15_N_3_O_4_S (309): C, 50.47; H, 4.89; N, 13.58; Found: C, 50.49; H, 4.90; N, 13.55.

### 3. Refinement

The amino H atoms were located on a difference map and isotropically refined. C-bound H atoms were geometrically positioned (C—H 0.95–0.99 Å), and refined using a riding model, with *U*_iso_(H) fixed to 1.2 – 1.5 × *U*(eq) of the parent atom.

### Crystal data

**Table:**

C_13_H_15_N_3_O_4_S	*F*(000) = 648
*M**_r_* = 309.34	*D*_x_ = 1.397 Mg m^−^^3^
Monoclinic, *P*2_1_/*n*	Melting point: 408 K
Hall symbol: P 2yn	Cu *K*α radiation, λ = 1.54184 Å
*a* = 6.27869 (7) Å	Cell parameters from 45300 reflections
*b* = 15.43607 (12) Å	θ = 4.1–75.6°
*c* = 15.27141 (13) Å	µ = 2.14 mm^−^^1^
β = 96.2633 (9)°	*T* = 100 K
*V* = 1471.24 (2) Å^3^	Column, colourless
*Z* = 4	0.25 × 0.12 × 0.10 mm

### Data collection

**Table:**

Oxford Diffraction Xcalibur (Atlas, Nova) diffractometer	3043 independent reflections
Radiation source: Nova (Cu) X-ray Source	3035 reflections with *I* > 2σ(*I*)
Mirror monochromator	*R*_int_ = 0.024
Detector resolution: 10.3543 pixels mm^-1^	θ_max_ = 75.8°, θ_min_ = 4.1°
ω scans	*h* = −6→7
Absorption correction: multi-scan (*CrysAlis PRO*; Agilent, 2012)	*k* = −19→19
*T*_min_ = 0.800, *T*_max_ = 1.000	*l* = −19→19
51079 measured reflections	

### Refinement

**Table:**

Refinement on *F*^2^	Secondary atom site location: difference Fourier map
Least-squares matrix: full	Hydrogen site location: inferred from neighbouring sites
*R*[*F*^2^ > 2σ(*F*^2^)] = 0.027	H atoms treated by a mixture of independent and constrained refinement
*wR*(*F*^2^) = 0.074	*w* = 1/[σ^2^(*F*_o_^2^) + (0.0381*P*)^2^ + 0.6757*P*] where *P* = (*F*_o_^2^ + 2*F*_c_^2^)/3
*S* = 1.05	(Δ/σ)_max_ = 0.002
3043 reflections	Δρ_max_ = 0.34 e Å^−^^3^
201 parameters	Δρ_min_ = −0.39 e Å^−^^3^
0 restraints	Extinction correction: *SHELXL97* (Sheldrick, 2008), Fc^*^=kFc[1+0.001xFc^2^λ^3^/sin(2θ)]^-1/4^
Primary atom site location: structure-invariant direct methods	Extinction coefficient: 0.0050 (4)

### Special details

**Table:**

Geometry. All e.s.d.'s (except the e.s.d. in the dihedral angle between two l.s. planes) are estimated using the full covariance matrix. The cell e.s.d.'s are taken into account individually in the estimation of e.s.d.'s in distances, angles and torsion angles; correlations between e.s.d.'s in cell parameters are only used when they are defined by crystal symmetry. An approximate (isotropic) treatment of cell e.s.d.'s is used for estimating e.s.d.'s involving l.s. planes.Least-squares planes (*x*,*y*,*z* in crystal coordinates) and deviations from them (* indicates atom used to define plane)2.2393 (0.0028) *x* + 11.4616 (0.0048) *y* - 9.2011 (0.0057) *z* = 0.8382 (0.0021)* 0.0093 (0.0008) C11 * -0.0049 (0.0008) C12 * -0.0055 (0.0009) C13 * 0.0116 (0.0008) C14 * -0.0075 (0.0008) C15 * -0.0030 (0.0008) C16Rms deviation of fitted atoms = 0.0076- 1.0360 (0.0033) *x* + 12.4363 (0.0050) *y* + 8.9114 (0.0069) *z* = 7.4119 (0.0015)Angle to previous plane (with approximate e.s.d.) = 77.48 (0.03)* 0.0208 (0.0006) N1 * -0.0138 (0.0006) N2 * 0.0016 (0.0007) C3 * 0.0109 (0.0007) C4 * -0.0196 (0.0006) C5Rms deviation of fitted atoms = 0.0150
Refinement. Refinement of *F*^2^ against ALL reflections. The weighted *R*-factor *wR* and goodness of fit *S* are based on *F*^2^, conventional *R*-factors *R* are based on *F*, with *F* set to zero for negative *F*^2^. The threshold expression of *F*^2^ > σ(*F*^2^) is used only for calculating *R*-factors(gt) *etc*. and is not relevant to the choice of reflections for refinement. *R*-factors based on *F*^2^ are statistically about twice as large as those based on *F*, and *R*- factors based on ALL data will be even larger.

### Fractional atomic coordinates and isotropic or equivalent isotropic displacement parameters (Å^2^)

**Table:**

	*x*	*y*	*z*	*U*_iso_*/*U*_eq_	
S1	0.23094 (4)	0.321249 (17)	0.345762 (16)	0.01627 (10)	
O1	0.02102 (13)	0.32316 (5)	0.37347 (6)	0.02126 (19)	
O2	0.41682 (14)	0.32016 (5)	0.40895 (5)	0.02177 (19)	
O3	0.69858 (13)	0.55623 (5)	0.15472 (5)	0.02182 (19)	
O4	0.39748 (12)	0.58590 (5)	0.06499 (5)	0.01882 (18)	
N1	0.25242 (14)	0.41272 (6)	0.28744 (6)	0.01595 (19)	
N2	0.06923 (14)	0.43553 (6)	0.23043 (6)	0.0178 (2)	
C3	0.14389 (17)	0.48527 (7)	0.17142 (7)	0.0168 (2)	
H3	0.0556	0.5119	0.1245	0.020*	
C4	0.36924 (17)	0.49552 (7)	0.18436 (7)	0.0156 (2)	
C5	0.43824 (17)	0.44590 (7)	0.25821 (7)	0.0156 (2)	
C6	0.50665 (17)	0.54783 (7)	0.13524 (7)	0.0158 (2)	
C7	0.52593 (19)	0.63686 (7)	0.01022 (7)	0.0201 (2)	
H7A	0.6350	0.6000	−0.0136	0.024*	
H7B	0.5999	0.6843	0.0449	0.024*	
C8	0.3728 (2)	0.67306 (8)	−0.06355 (8)	0.0272 (3)	
H8A	0.2989	0.6253	−0.0966	0.041*	
H8B	0.4525	0.7073	−0.1031	0.041*	
H8C	0.2672	0.7101	−0.0390	0.041*	
C11	0.24897 (18)	0.23989 (7)	0.26731 (7)	0.0173 (2)	
C12	0.07233 (19)	0.22397 (8)	0.20604 (8)	0.0219 (2)	
H12	−0.0601	0.2526	0.2104	0.026*	
C13	0.0935 (2)	0.16557 (8)	0.13850 (8)	0.0248 (3)	
H13	−0.0258	0.1540	0.0964	0.030*	
C14	0.2884 (2)	0.12352 (8)	0.13168 (8)	0.0233 (3)	
C15	0.46013 (19)	0.13919 (8)	0.19509 (8)	0.0227 (2)	
H15	0.5914	0.1094	0.1917	0.027*	
C16	0.44368 (18)	0.19753 (8)	0.26327 (8)	0.0200 (2)	
H16	0.5622	0.2083	0.3061	0.024*	
C17	0.3134 (3)	0.06341 (9)	0.05575 (9)	0.0335 (3)	
H17A	0.3628	0.0964	0.0070	0.050*	
H17B	0.1751	0.0364	0.0362	0.050*	
H17C	0.4185	0.0184	0.0747	0.050*	
N3	0.63513 (16)	0.43168 (7)	0.29848 (7)	0.0214 (2)	
H02	0.743 (3)	0.4452 (11)	0.2705 (11)	0.033 (4)*	
H01	0.655 (3)	0.3949 (11)	0.3402 (11)	0.033 (4)*	

### Atomic displacement parameters (Å^2^)

**Table:**

	*U*^11^	*U*^22^	*U*^33^	*U*^12^	*U*^13^	*U*^23^
S1	0.01694 (16)	0.01813 (15)	0.01412 (15)	0.00056 (9)	0.00335 (10)	0.00249 (9)
O1	0.0211 (4)	0.0234 (4)	0.0207 (4)	0.0005 (3)	0.0088 (3)	0.0033 (3)
O2	0.0227 (4)	0.0262 (4)	0.0158 (4)	−0.0007 (3)	−0.0003 (3)	0.0038 (3)
O3	0.0144 (4)	0.0265 (4)	0.0243 (4)	−0.0008 (3)	0.0014 (3)	0.0056 (3)
O4	0.0169 (4)	0.0225 (4)	0.0170 (4)	0.0001 (3)	0.0019 (3)	0.0058 (3)
N1	0.0135 (4)	0.0179 (4)	0.0166 (4)	0.0008 (3)	0.0021 (3)	0.0024 (3)
N2	0.0137 (4)	0.0199 (5)	0.0196 (5)	0.0021 (3)	0.0007 (3)	0.0024 (4)
C3	0.0157 (5)	0.0177 (5)	0.0172 (5)	0.0015 (4)	0.0022 (4)	0.0008 (4)
C4	0.0149 (5)	0.0166 (5)	0.0155 (5)	0.0013 (4)	0.0021 (4)	0.0000 (4)
C5	0.0153 (5)	0.0168 (5)	0.0150 (5)	−0.0003 (4)	0.0036 (4)	−0.0014 (4)
C6	0.0164 (5)	0.0156 (5)	0.0154 (5)	0.0023 (4)	0.0025 (4)	−0.0003 (4)
C7	0.0248 (6)	0.0183 (5)	0.0180 (5)	−0.0018 (4)	0.0065 (4)	0.0025 (4)
C8	0.0379 (7)	0.0241 (6)	0.0196 (6)	0.0024 (5)	0.0029 (5)	0.0056 (4)
C11	0.0194 (5)	0.0161 (5)	0.0168 (5)	0.0014 (4)	0.0040 (4)	0.0028 (4)
C12	0.0203 (6)	0.0217 (6)	0.0235 (6)	0.0034 (4)	0.0007 (4)	0.0006 (4)
C13	0.0291 (6)	0.0235 (6)	0.0210 (6)	0.0021 (5)	−0.0015 (5)	0.0005 (5)
C14	0.0346 (7)	0.0171 (5)	0.0194 (6)	0.0023 (5)	0.0084 (5)	0.0040 (4)
C15	0.0247 (6)	0.0191 (5)	0.0258 (6)	0.0059 (4)	0.0101 (5)	0.0063 (4)
C16	0.0188 (5)	0.0200 (5)	0.0215 (6)	0.0021 (4)	0.0033 (4)	0.0057 (4)
C17	0.0525 (9)	0.0260 (6)	0.0236 (6)	0.0062 (6)	0.0116 (6)	−0.0008 (5)
N3	0.0149 (5)	0.0295 (5)	0.0196 (5)	−0.0002 (4)	0.0011 (4)	0.0078 (4)

### Geometric parameters (Å, º)

**Table:**

S1—O1	1.4279 (8)	C14—C15	1.3898 (18)
S1—O2	1.4311 (8)	C14—C17	1.5065 (17)
S1—N1	1.6827 (9)	C15—C16	1.3890 (17)
S1—C11	1.7478 (11)	C3—H3	0.9500
O3—C6	1.2163 (14)	C7—H7A	0.9900
O4—C6	1.3437 (13)	C7—H7B	0.9900
O4—C7	1.4546 (13)	C8—H8A	0.9800
N1—C5	1.3915 (14)	C8—H8B	0.9800
N1—N2	1.4093 (12)	C8—H8C	0.9800
N2—C3	1.3089 (14)	C12—H12	0.9500
C3—C4	1.4160 (15)	C13—H13	0.9500
C4—C5	1.3927 (15)	C15—H15	0.9500
C4—C6	1.4492 (15)	C16—H16	0.9500
C5—N3	1.3375 (14)	C17—H17A	0.9800
C7—C8	1.5067 (16)	C17—H17B	0.9800
C11—C12	1.3927 (16)	C17—H17C	0.9800
C11—C16	1.3937 (16)	N3—H02	0.865 (18)
C12—C13	1.3870 (17)	N3—H01	0.852 (18)
C13—C14	1.3990 (18)		
			
O1—S1—O2	120.77 (5)	C15—C16—C11	118.34 (11)
O1—S1—N1	105.63 (5)	N2—C3—H3	123.3
O2—S1—N1	105.08 (5)	C4—C3—H3	123.3
O1—S1—C11	110.42 (5)	O4—C7—H7A	110.5
O2—S1—C11	110.17 (5)	C8—C7—H7A	110.5
N1—S1—C11	103.01 (5)	O4—C7—H7B	110.5
C6—O4—C7	115.40 (8)	C8—C7—H7B	110.5
C5—N1—N2	111.48 (8)	H7A—C7—H7B	108.6
C5—N1—S1	126.66 (8)	C7—C8—H8A	109.5
N2—N1—S1	115.43 (7)	C7—C8—H8B	109.5
C3—N2—N1	104.03 (9)	H8A—C8—H8B	109.5
N2—C3—C4	113.39 (10)	C7—C8—H8C	109.5
C5—C4—C3	105.62 (9)	H8A—C8—H8C	109.5
C5—C4—C6	125.13 (10)	H8B—C8—H8C	109.5
C3—C4—C6	129.22 (10)	C13—C12—H12	120.6
N3—C5—N1	123.87 (10)	C11—C12—H12	120.6
N3—C5—C4	130.76 (10)	C12—C13—H13	119.6
N1—C5—C4	105.34 (9)	C14—C13—H13	119.6
O3—C6—O4	123.66 (10)	C16—C15—H15	119.3
O3—C6—C4	124.24 (10)	C14—C15—H15	119.3
O4—C6—C4	112.10 (9)	C15—C16—H16	120.8
O4—C7—C8	106.37 (9)	C11—C16—H16	120.8
C12—C11—C16	121.71 (11)	C14—C17—H17A	109.5
C12—C11—S1	118.75 (9)	C14—C17—H17B	109.5
C16—C11—S1	119.35 (9)	H17A—C17—H17B	109.5
C13—C12—C11	118.71 (11)	C14—C17—H17C	109.5
C12—C13—C14	120.83 (11)	H17A—C17—H17C	109.5
C15—C14—C13	119.07 (11)	H17B—C17—H17C	109.5
C15—C14—C17	120.36 (12)	C5—N3—H02	117.9 (11)
C13—C14—C17	120.56 (12)	C5—N3—H01	120.2 (11)
C16—C15—C14	121.31 (11)	H02—N3—H01	118.3 (15)
			
O1—S1—N1—C5	169.64 (9)	C5—C4—C6—O3	−2.20 (18)
O2—S1—N1—C5	40.91 (10)	C3—C4—C6—O3	175.58 (11)
C11—S1—N1—C5	−74.48 (10)	C5—C4—C6—O4	177.48 (10)
O1—S1—N1—N2	−41.11 (9)	C3—C4—C6—O4	−4.74 (16)
O2—S1—N1—N2	−169.84 (7)	C6—O4—C7—C8	−179.98 (9)
C11—S1—N1—N2	74.78 (8)	O1—S1—C11—C12	36.77 (10)
C5—N1—N2—C3	−3.39 (12)	O2—S1—C11—C12	172.69 (9)
S1—N1—N2—C3	−157.24 (8)	N1—S1—C11—C12	−75.64 (10)
N1—N2—C3—C4	1.50 (12)	O1—S1—C11—C16	−148.21 (9)
N2—C3—C4—C5	0.83 (13)	O2—S1—C11—C16	−12.29 (11)
N2—C3—C4—C6	−177.28 (11)	N1—S1—C11—C16	99.39 (9)
N2—N1—C5—N3	−177.82 (10)	C16—C11—C12—C13	−1.26 (17)
S1—N1—C5—N3	−27.56 (16)	S1—C11—C12—C13	173.65 (9)
N2—N1—C5—C4	3.92 (12)	C11—C12—C13—C14	−0.18 (18)
S1—N1—C5—C4	154.18 (8)	C12—C13—C14—C15	1.74 (18)
C3—C4—C5—N3	179.10 (12)	C12—C13—C14—C17	−177.06 (11)
C6—C4—C5—N3	−2.68 (19)	C13—C14—C15—C16	−1.94 (17)
C3—C4—C5—N1	−2.81 (12)	C17—C14—C15—C16	176.87 (11)
C6—C4—C5—N1	175.41 (10)	C14—C15—C16—C11	0.56 (17)
C7—O4—C6—O3	2.02 (15)	C12—C11—C16—C15	1.07 (17)
C7—O4—C6—C4	−177.65 (9)	S1—C11—C16—C15	−173.81 (8)

### Hydrogen-bond geometry (Å, º)

**Table:**

*D*—H···*A*	*D*—H	H···*A*	*D*···*A*	*D*—H···*A*
N3—H02···O3	0.865 (18)	2.456 (17)	2.9775 (13)	119.4 (13)
N3—H01···O2	0.852 (18)	2.237 (17)	2.8624 (13)	130.3 (14)
N3—H02···N2^i^	0.865 (18)	2.206 (18)	3.0216 (14)	157.1 (15)
N3—H01···O1^i^	0.852 (18)	2.554 (17)	3.0631 (13)	119.3 (13)

Symmetry code: (i) *x*+1, *y*, *z*.
